# Culture of integrity – institutional response to integrity during COVID19

**DOI:** 10.1007/s40979-022-00118-9

**Published:** 2022-11-28

**Authors:** Zeenath Reza Khan, Joice Priya, Christopher Tuffnell

**Affiliations:** University of Wollongong in Dubai, PO Box 20183, Knowledge Park, Dubai, UAE

**Keywords:** COVID19, Academic integrity, Institutional response, Assessment design, Policy framework

## Abstract

COVID19 forced most education institutions to move their education provisions to digital platforms almost overnight to ensure continued learning for students. Variable factors relating to educational technologies, ease of use, accessibility and funding meant the process was more challenging for some than others. However even the most agile and well-prepared educational institutions faced a hurdle during remote learning when it came to integrity in assessing students online.

This article tracks the efforts by one western university in a Middle Eastern country, tracing back the systemic changes, evolution of policies and procedures that culminated into a campus-wide response that helped redesign assessments, increased awareness among faculty towards recognising and reporting cases, and the implementation of integrity pledges. It is believed this article presents a well-rounded case study as a good practice guide for other tertiary institutions on the importance of building a culture of integrity prior to any crisis, that can help whether such situations need to arrive in the future.

## Introduction

Around the world, March 2020 became synonymous with a shift to remote learning or more aptly named emergency distance learning (EDL) as countries went into lock down, schools and universities shut their doors, and more than 1.5 billion students were suddenly out of brick and mortar classrooms across 144 countries (UNESCO 2020). This meant students across nations were suddenly at home and online. Government entities, non-government entities and education institutions worked together to move learning to digital platforms for the provision of EDL. EDL can be defined as a “temporary shift of instructional delivery to an alternate delivery mode due to crisis circumstances” (Hodges et al. 2020, para 13). EDL is a means to help continue education for students in the face of adversities. Prior to the 2020 pandemic, we saw such examples in Hong Kong amidst protests where universities began distance learning (Lau 2019).

The shift to EDL can come with its own set of challenges, particularly in academic integrity during assessments: lack of preparation, infrastructure, achievement of learning outcomes, commitment of students and most dramatically, the issue of academic dishonesty (Guangul, et al 2020). Institutions such as Texas Agricultural and Mechanical College of Texas (Texas A&M) have investigated “large scale” cheating, particularly through sites such as Chegg.com – a file sharing site that borders on being a contract cheating service (McGee 2020); to United States (US) Military Academy at West Point’s accusation of more than 70 cadets cheating online (Brook 2020); to the polarised arguments for and against proctoring services for online exams (Asher-Schapiro 2020); the issues are not new, but came into even greater scrutiny during EDL.

Amidst these challenges, this paper traces the efforts by one branch campus of a western university in the Middle East that highlights the importance of a decade-long, proactive effort in developing a culture of integrity across the campus that invariably helped the institution face the above-mentioned challenges during the pandemic’s EDL. The authors, who include faculty, QA unit member and instructional designer, posit this case study to be a good practice example for quality-assurance staff, university executives, academic and education boards and policymakers in highlighting how holistic approach to systematic review of policies, regular awareness campaigns and training sessions among others can help universities without having to reinvent the wheel in times of crises. We position our narrative and interpretations presented in this paper as stakeholders from different areas, disciplines and levels of a university, with understandable bias as those working in academic institutions with pluralistic views and approaches to academic integrity.

The paper is organised as follows: it begins with a brief overview of academic integrity in online platforms, then introduces the work done on academic integrity in the United Arab Emirates. The paper then introduces the case campus and traces its efforts in terms of policy and governance, awareness campaigns, research conducted, and support provided to students and faculty before and during the pandemic. The paper discusses the efforts in terms of number of cases reported and concludes the impact of the institutional response to COVID19 EDL.

## Academic integrity

Academic Integrity is the foundation of education. The International Centre for Academic Integrity identifies six fundamental values as defining academic integrity—honesty, fairness, truth, respect, responsibility, and courage (ICAI 2013). Different types of actions have been identified as breaches of academic integrity. Newstead et al. (1996) identified 21 such behaviours that can be considered as cheating such as cheating in exams, impersonating someone else to sit for exams, using unauthorised material during exams, collusion, fraud, impersonating others, using someone else’s work as their own, paying someone to get their own work done and so on; while Khan (2014) added “electronic cheating”, and others looked at assessment design and more (Harmon, Lambrinos and Buffolino 2010; Bretag et al. 2018; Harrison 2020).

For decades higher education academics and researchers have grappled with ways to tackle and curb student misconducts. McCabe’s decades-long study of over 70,000 students in the USA found that 95% of students admitted to having engaged in some form of cheating (ICAI 2020). While this has varied slightly over the years in the range of 75—85% (McCabe & Trevino 1993; Genereux & McLeod 1995; Ekstein 2003; Hemby, Wilkinson & Crews 2006; King et al. 2009; Tanner & Piper 2010), most recent studies have also indicated similar self-reported cases globally (Farkas 2017; Newton 2019; Schaffhauser 2020).

Academic misconduct has a severe and long-lasting impact on universities. The 2012 Harvard School’s scandal in US that identified approximately 125 students suspected of cheating in a take-home exam (Pérez-Peña and Bidgood, 2012a, b), the 2015 contract cheating scandal that impacted more than a dozen universities in Australia (Bretag 2015), the 2019 US admission scandal involving Hollywood parents that shook the Ivy league schools (Durkin 2019) or the West Point cadets cheating in the US (AP 2020) all have showed the detrimental and lasting impact of such cases from losing reputation to student anxiety to difficulties in graduation, employability, admissions and more (Levrik 2012). This paper focuses on the Middle East where studies have shown a similar number of cases reported by faculties and students on assessments and exams (McCabe et al. 2008; Khan and Subramanian 2012; Mullan 2016; Khan et al. 2019).

While some studies have highlighted the importance of assessment design, teaching modules, teachers’ perceptions, policies, penalty and detection, and other areas as crucial in deterring misconduct behaviours; other studies have posited on the importance of developing a culture of integrity and depending on that culture to help universities manage students’ likelihood to cheat or e-cheat (Khan 2014; Khan and Subramanian 2012; Peters 2019).

Morris and Carroll (2016) talked extensively about focusing on staff to overcome challenges in effectively implementing academic integrity policies and procedures. Orr (2018) focused on an educational seminar to help transition from punitive systems to educational one; while Cronan et al. (2017) presented a case for technology-based intervention to help change knowledge and attitudes of students. Hudd et al. (2009) talked about the importance of focusing on full time and part time faculty; Morrow (2022) spoke on the importance of librarians’ roles as advocates for academic integrity; Thacker & McKenzie (2022) highlighted the need for quality assurance frameworks that can support culture of academic integrity; Betram-Gallant & Drinan (2006) placed the importance on the administrators of universities, rather than on students, concluding the need to groom faculty as agents of change in developing a culture of integrity.

The above list is by no means exhaustive; but we believe it provides precedence for the gap in literature. Hendershott et al. (2000) posited that there is a “need to involve every layer of an institution, including students, faculty, administrators, and governing boards…to the creation of a culture that will support and sustain a climate of academic integrity” (p. 587). Similarly, Donald McCabe (2005) argued that although honour codes work, they are not enough; what matters is the organisational culture students come into when they join universities, a view that has been supported extensively in literature (Devlin 2002; Park 2003; Hulsart & McCarthy 2011). McCabe went on to list three strategies that encapsulated the need to ensure clarity from faculty on their expectations of academic conduct, administrators’ role in policy review and management’s need to focus also on students who had offended (2005).

There needs to be a good practice case on what a holistic approach may look like at a university, and more so how that approach may help the university in times of crises. Of particular interest to this paper is the United Arab Emirates (UAE)’s western campus University of Wollongong in Dubai (UOWD) that has been pioneering research and awareness initiatives in the Gulf, with regular presence at the International Day of Actions against Contract Cheating events and so on. This paper is an attempt at narrating longitudinal efforts across a decade by UOWD, using scientific, qualitative methodology to record efforts that the university has made in developing a culture of integrity and how such systematic efforts helped weather the emergency distance learning and online assessment challenges when it comes to upholding academic integrity.

### COVID19 pandemic and academic integrity

COVID19 has brought about some unprecedented challenges to the higher education sector globally. With millions of students unable to attend on-campus, face to face classes, universities were forced to move teaching and learning to online platforms as Governments took rapid, high-stakes action in response to the global COVID-19 Pandemic. The UAE was no exception, and precautions were taken across the seven Emirates that affected all sectors. The UAE Ministry of Education (MoE) was quick to transition, as early as March 2020 (MOE 2020a) giving institutions weeks (adjusting Spring breaks) to prepare and then begin online classes. Policies addressing how institutions were to move to distance learning, requirements for assessment procedures, teacher and staff training and others were drafted and disseminated with the goal of protecting students and institutional staff whilst also aiming for the continuation of education, albeit delivered differently (MOE 2020b).

The MoE referred to the fully online mandated approach as distance learning. However, the experience of faculty and students during this period could potentially cast a negative light on the concept of distance or online learning due to the rapid, unplanned nature of the transition; in view of this, the case University referred to this period as emergency distance learning (EDL) to differentiate from that of well-designed distance education.

Globally, immediate focus was on the delivery of content in the new online modality; however, soon, the issue of integrity when delivering the online lessons and assessing students in a digital platform came to the forefront. From collusion in group chats for the same course to sharing answers during tests (Haney 2020), to imposters taking online exams (Newton 2020), to using online answer or essay writing sites (Newton 2020) and more, universities were grappling with misconduct issues. In response of course, some universities used proctoring services online which have brought their own share of issues pertaining to student privacy (Morrison & Jeilweil 2020), while others revisited assessment questions and redesigned how they assessed their students (Bretag et al. 2019).

However, the fact remains that the pandemic has held up a mirror to institutions, forcing universities to look within their systems, policies, approaches to teaching, assessing, detection and more to see how they can instil the right kind of values in students that would remain relevant when students move from physical classrooms to the virtual ones, thus changing, in many cases, the culture across the university systems.

### Research objective

The pandemic is far from being over. Many countries are facing waves of infection, with the number of infected constantly fluctuating (Kluge 2022). New strains are still being discovered (ECDC 2022), while there remains a growing threat from other diseases such as the Monkeypox which has made its presence felt for the better part of 2022 (WHO 2022). Other extenuating circumstances such as the Ukraine war of 2022 (Este 2022), Hong Kong protests in 2019 (Reuters 2020) and even the Afghanistan war of 2001 (Witte n.d.) have led to discontinuity of education for students. While Ukraine war is still on-going during the finalising stages of this manuscript, events such as the Hong Kong protests and the Afghanistan war have presented case studies of distance learning through television (IWPR, 2007), e-learning alliances with government and non-government agencies such as United States Agency for International Development (USAID)’s Afghan e-Quality Alliance Program (Beebe 2010), and online learning programmes (Allan 2020).

Distance learning itself has been around for three centuries (Pappas 2013); and studies have constantly highlighted concerns surrounding academic cheating (Khan and Samuel 2009; Khan 2009; King et al. 2009; VilChez 2011; Raines, Ricci & brown 2011; Khan and Balasubramanian 2012; Bemmel 2014; Abazorius 2015).

The question that this paper then tries to answer is – how does a university campus manage and maintain integrity in assessments beyond traditional classrooms, while conducting emergency distance learning?

This paper presents a reflective case study of the case University’s systematic efforts over a decade as proactive actions from grassroots to policy and procedures that culminated in helping them respond to the pandemic in upholding integrity of their courses, rather than patch-work efforts in the face of emergency distance learning.

### Research methodology

This paper uses a narrative story-telling methodology to present the case study of one university’s response to the pandemic and how it tackled the issue of upholding academic integrity online. Narrative story telling has been recognised as an established method of qualitative research (Kendall and Kendall 2012; Lumsden 2018; Lewis and Hildebrandt 2019). This is because it allows for in-depth understanding and exploration of attitudes, behaviour, actions, and steps taken, barriers and opportunities (McCall et al. 2021). Potts (2004) posited that in fact storytelling is a qualitative tool that allows for “self-examination, reconstruction of memory, and construction of meaning” (p17).

The authors attempted to build the narrative case through archival evidence, ethnographic and lived experiences of the authors at the University. Ethnography is a research methodology that allows us to observe a cultural setting and produce a narrative account (Lindlof & Taylor 2002; Whitehead 2005). Whitehead (2005) goes on to express how ethnography is a “process of discovery, making inferences, and continuing inquiries in an attempt to achieve emic validity” (p. 4). This is vital to our research and the subsequent findings we present because ours is considered “native’s point of view” as we are immersed in the culture of the University as faculty and staff members (Malinowski, 1922, p. 25).

We have further triangulated the observations for reliability (Denzin 1975; Rock 2001; Tiainen & Koivunen 2006), with our own informal experiences and multiple data sources such as:

- observations of processes that were put in place in response to EDL
- reviewing of implementation of such processes through archival documents such as evaluation report, feedback reports provided by QA unit
- reviewing of documentation and reports that were prepared for training, informational or for recording of the processes and changes brought about at the University either before or during the pandemic that were at various levels: subject level, degree/department level and institutional level.

The team of authors represented different stakeholder groups (faculty, instructional designers, quality assurance staff) and included informal discussions with key stakeholders (faculty, management staff) from the quality assurance department, the ad hoc and informal blended learning team (communities of practice) formed by various volunteer academics who provided training and workshops for other faculty during the pandemic and through review of policies and procedures implemented by the University in the decade leading to and through the pandemic. Some of the artefacts examined indirectly or directly included, but were not limited to, quality assurance reports, student evaluation and feedback reports, academic integrity and misconduct reports, policy documents on course assessments, academic integrity and misconducts, and others.

The study did not include direct human participants, using mainly secondary data, therefore received necessary exemptions and approval of use from the custodians of data at the University, and an ethical clearance from the respective committees.

### Case focus—the University

An Australian university’s branch campus or partner university in the Middle East, UOWD is one of the longest running private, western universities in the UAE. The University offers 45 programs (both undergraduate and postgraduate) in the areas of Business, Engineering, Information Sciences, Humanities, Social Sciences and Health. In addition, the University offers a doctoral program in business and offers via the College—English Language pathways programs, Global English skills etc. The University on an average has around 3000 students who represent more than 108 nationalities and staff represent around 50 nationalities. Most students are from the subcontinent (India, Pakistan, Bangladesh). UAE nationals, Emirati students, form around 10–13% of the student population. The University has more than 11,000 alumni.

### UOWD and Quality Assurance (QA)

**Fig. 1:**
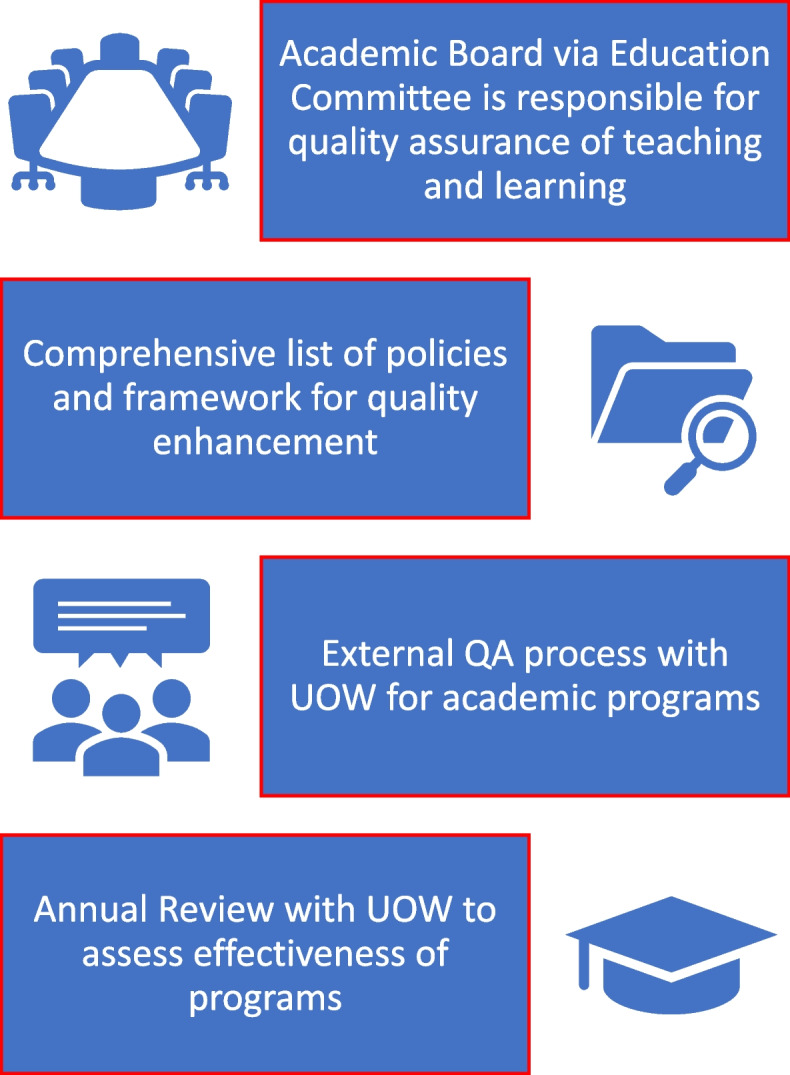
Quality and Continuous improvement at UOWD

Due to the University’s long-standing history and being a partner university, a culture has been established that recognises the importance of quality, and the continuous improvement processes. It follows a mature approach to governance and robust quality assurance processes that ensure that education quality is maintained consistently across all faculties. This is in keeping with findings from Wilkins (2010), Knight (2011) and particularly Karam (2018) which focuses on the Middle East that suggest international branch campuses can maintain the brand and quality of the programs offered in primary provider campus with integrity, balance independence and partnership with primary provider and at the same time align themselves with national priorities of home and host countries. For UOWD, this is illustrated in Fig. 1:

The governance structure follows a unitary governance model that is structured about boards that are responsible for strategic institutional decisions, particularly due to the overall size of the University (Pruvot & Estermann 2018).

The University’s Academic Board via the Education Committee has ultimate responsibility for quality assurance (QA) of teaching and learning activities across the University and is supported by the role of Associate Dean Education (ADE) in each faculty. The University has a comprehensive list of policies and a framework that facilitates continuous quality enhancement across all its programs. Some of the key features of QA include:

(i) annual review of the program including performance (Kinser & Lane 2017),
(ii) assessment committee meetings where student performance and delivery of the subject are discussed in comparison to previous sessions. This also includes a self-reflection from the lecturers identifying the strengths and areas for improvements of their presentation of the subjects in terms of an individual subject (Rasmussen 1997; Brennan & Shah 2000),
(iii) internal QA process (Vroeijenstijn 1995; Finch 1997) – where a faculty member with expertise in the subject assesses that the (a) subject content and assessments are appropriate to the stated student outcomes of the subject, (b) contain assessment tasks which are fair, appropriate to the level of the subject and answerable.

The University, which is a partner university, faces an extensive external QA process with the primary provider (University of Wollongong (UOW)) to provide external review and continuous improvement cycle for all academic programs offered by UOWD annually. This process also provides a unique opportunity for benchmarking between UOWD and primary provider which contributes to verification and consistency of academic standards, thus ensuring integrity as posited by Kis (2005) and Wolff (2015). In keeping with findings from Hou et al. (2018) such QA processes allow for a comprehensive review and analysis of all academic matters once per year, helping to check and maintain integrity in teaching, learning, and assessing. This includes evaluation of the QA process and other issues relevant to maintaining/expanding the delivery of high-quality teaching programs at UOWD. Matters discussed in detail include student progression and achievement, subjects with high failure rates, student learning experience, student academic support, teaching and learning resources, assessment and feedback, academic integrity, grievances, adequacy of faculty workload, program reviews, approvals and accreditation requirements and compliance and curriculum changes.

### UOWD and academic integrity

In this section, we attempt to track the teaching, learning, assessment, and research work that the University has engaged in across all disciplines, departments and partnering with all stakeholders aligning with Eaton (2021)’s 4 M (mega-macro-meso-micro) framework, grounded in the argument that multiple stakeholders are to be held responsible for fostering a culture of integrity at a university (Betram-Gallant; 2008).

#### Research

 The University has been a pioneer in researching academic integrity in the region. Faculty have been publishing in the area since 2006 such as Khan (2007), Khan and Samuel (2009), Khan (2009), Khan and Balasubramanian (2012), Khan (2010) and so on (see Table 1 Appendix for detailed list of publications). Furthermore, the University applied to host two international conferences. The first was the International Conference on Academic Integrity—Middle East Chapter, in collaboration with Centre for Academic Integrity and Clemson University in the US (Emirates 24/7 2016) where 60 local and international delegates attended, 16 papers were presented, two keynote speakers international renowned joined and conducted workshops as well for the universities and schools in the UAE (Khan, 2016). The second was the 6th International Conference Plagiarism Across Europe and Beyond 2020 that the University bid to win hosting privileges for European Network for Academic Integrity (UOWD 2020). This conference hosted four keynote speakers, more than 100 delegates (local and international), 35 paper presentations, 10 workshops and three poster sessions. While the 2016 conference had only one paper presentation from the University, the 2020 conference had 10 paper and workshop presentations (Hill, Khan & Kralikova 2020).

The University funds heavily on projects related to different facets of academic integrity, with the primary provider granting funds for additional large-scale, international projects. One PhD has been successfully completed by staff from the University under full scholarship from the primary provider (Khan 2014), while there is one under way at the time of writing this paper.

**Fig. 2:**
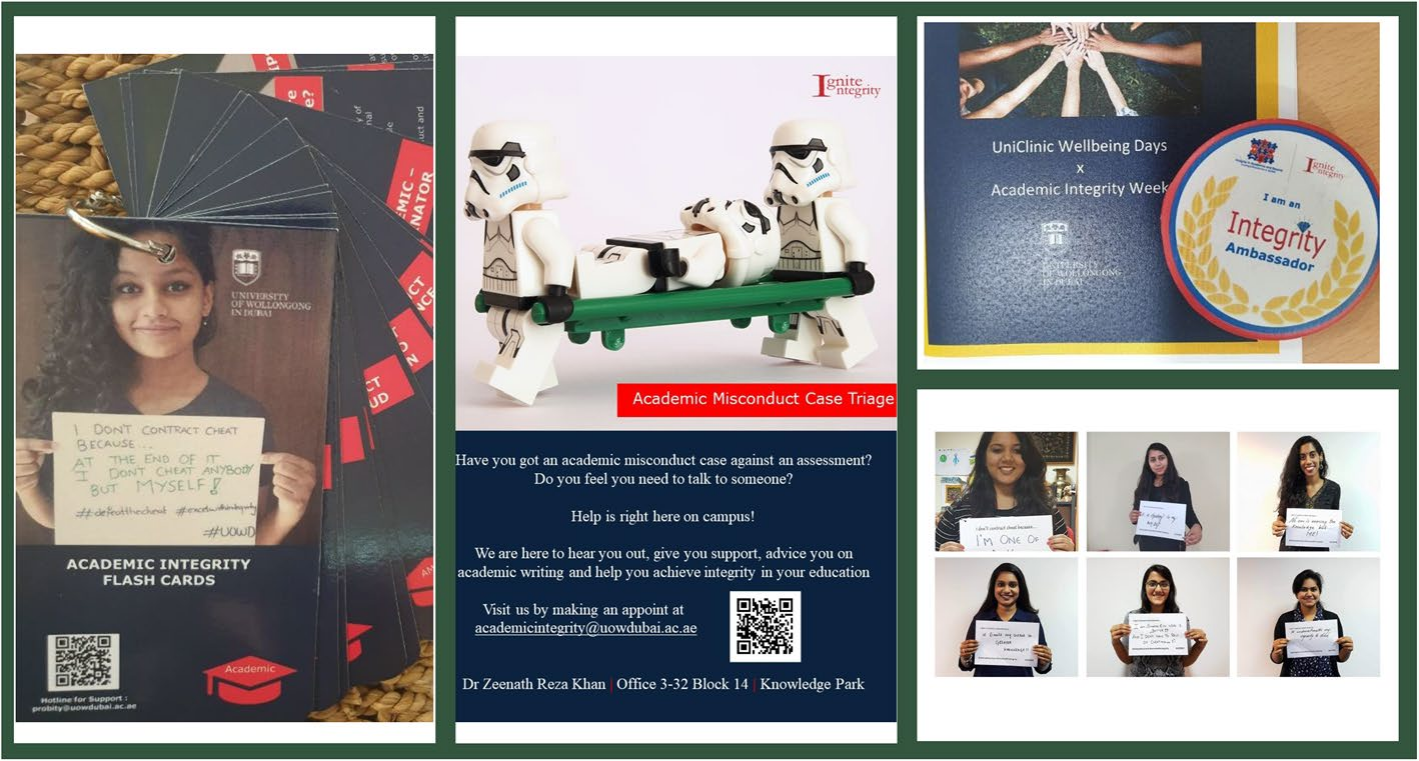
On-campus efforts and campaigns to foster culture of integrity (images presented with consent)

Faculty working in the area received research grants that helped develop flash cards and organise roadshows to further continue awareness campaigns (see Fig. 2).

In 2020, the university also supported and became host campus to the Centre for Academic Integrity in the UAE, first such national-level, voluntary endeavour that is composed of faculty, teachers and students from numerous schools and universities in the UAE (Hill & Khan 2021).

#### Assessment design and structure

Academic Integrity and quality assurance are integral elements of the standards set by Tertiary Education Quality and Standards Agency in Australia (TEQSA) and the local regulator in the UAE, i.e. Commission for Academic Accreditation (CAA;) and are the focus of scrutiny by these accrediting bodies. The University is mandated to demonstrate evidence of oversight of academic and research integrity and to have appropriate measures in place to ensure that design of assessments support the development of the skills necessary to demonstrate academic integrity and minimise opportunities for academic misconduct.

Over the years, the University has continuously improved upon its assessment practices to ensure that the university has in place quality assurance processes and procedures to support effective teaching and appropriate, consistent and fair assessment practices. Text-based assessment submissions have been submitted through text-matching software since the early 2000s.

Of particular interest is the year when we began looking closely at assessment design in relation to academic integrity—2012. We began moving away from practices such as large-scale use of multiple-choice questions, re-using exam questions; and observing practices such as making past papers available via library repository to making questions more application based and reflective. Since then, there has been a strong focus on the alignment of assessment tools and class activities to objectives of individual subjects to ensure the achievement of learning outcomes upon successful completion of subjects.

We observe that the year mentioned above coincides with the much-publicised Harvard Cheating Scandal (Pérez-Peña & Bidgood, 2012) that was reported around the summer of 2012 where about 125 students were found to have cheated on take-home exams. As academic and staff involved with education committee and faculty-level committees at the time, we remember how this was a point of discussion, highlighting the importance of looking at assessment design and academic integrity; a behaviour we have also seen recorded in other universities, how scandals have been catalysts to developing takeaways and lessons (Grunfeld 2012; Wheeler 2018; Stevens 2019).

**Fig. 3:**
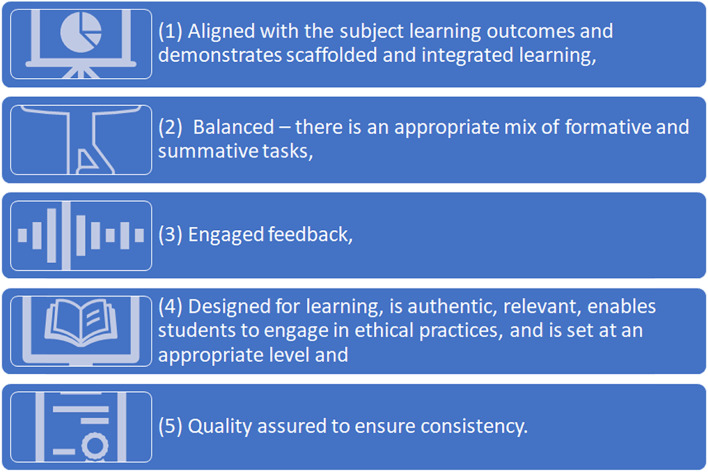
Five principles that govern assessment design at UOWD

The University’s policy on assessment ensures that design of assessments is based on the following principles (see Fig. 3):

Grounded in research and existing literature that draw a clear link between assessment design and academic integrity (Khan et al. 2021, Ellis et al. 2020; Sotiriadou et al. 2019; Hamilton & Richardson 2007; Egan 2018; Mellar et al. 2018; Rogerson 2017; Baird & Clare 2017), the University has been conscious about the over assessment of students using quizzes and tests, and have introduced other initiatives to enhance the teaching and learning experiences. These included integration of more real-world experiences for students in the form of capstone projects, introduction of reflective projects, internships, and essays. Group projects required students to provide individual reports and sometimes included a viva to ensure the integrity of the assessment process, and contributions.

#### Policies and procedures

Beyond the assessment design, the University’s policies and procedures also underwent review through rounds of focus group interviews with staff, faculty, and students between 2015–2016. Once again, we believe the timeline is very important and noteworthy. As an Australian partner university, we were very aware of the 2015 MyMaster contract cheating scandal that hit 16 universities in the country, impacting 1000 students leading to implications including revoking of degrees (Kennedy 2015; Visentin 2015). In the wake of the scandal, under the mandate of TEQSA (2017), like many universities in Australia, the primary provider rolled out a university-wide policy review process which trickled down to the partner university. This included conducting focus-group interviews with faculty and students, reviewing procedures of reporting (which was manual at this point), reviewing definitions, approach and more to revise the policy as per the Academic Integrity Standards Project (AISP 2012).

In coordination with the primary provider, the policies were updated and made more educative, with clear guidelines on the process of detecting and registering allegations of misconduct following guidelines called AWARE as proposed by Rogerson (2016) that focused on raising awareness, educating staff and students, etc. Online reporting system was introduced along with an Academic Integrity Learning Module (developed by the primary provider) to help students learn and rehabilitate. Furthermore, the detection and allegation reporting structure was streamlined, and Academic Integrity Officers (AIO) introduced in all faculties who underwent training and completed the Learning Module.

The above changes were rolled out in phases, with training and workshops held for faculty and students. This saw an increase in faculty uptake of the policy and procedures as faculty began to use the reporting system.

Furthermore, from 2019 specific academic integrity values workshops were introduced for new students joining the University at all levels, the merits of such initiatives were rooted in literature such as Lowe et al. (2018), Benson et al. (2019). Particularly, in recognition of the gap in prior knowledge, that new students came with depending on the schooling system they had graduated from, that may lead to students committing misconduct in the first year (Denisova-Schmidt 2016), the workshop designed was to ensure students understood the academic integrity values, were aware of the policies and were introduced to the basics of academic writing, and developed a view of the University as a campus that values integrity (McCabe 2005; Hulsart & McCarthy 2011).

Recognising the impact of detection and penalty on students (Olafson et al. 2013; Ballantine et al. 2018; Rabbi 2018), the University implemented a Triage clinic in 2019 as a support system for students who had faltered to help with the rehabilitation process and help reduce repeat offences (Khan et al. 2020c).

#### Student involvement

Hill (1995) argued for the need to include students in upholding quality of education. Devlin (2002), MacDonald & Carroll (2006) and others such as Freeman et al. (2007) have all posited that a holistic approach that includes students can help in tackling academic integrity issues. More recently, Khan (2021) argued for inclusion of students as partners rather than as receivers of messages when talking about academic integrity. From investigative methods where studies have shown the need to move from self-reporting to case study methods in capturing student attitudes and behaviour (Dawson & Overfield 2006); to involving students as ambassadors and forming societies on academic integrity (Richardson et al. 2016), one driving indicator for the University in developing a culture of integrity on campus has been to look at how students could be included as partners.

For the 2016 conference organised, the organising committee included students who were interested in helping to make decisions about the organisation of an event. In 2016, when the Centre for Academic Integrity announced the Global Ethics Day (third Wednesday of October) as the International Day of Action against Contract Cheating, the university registered a celebration that evolved into Week of Actions against Academic Misconduct (WAAAM) from 2017. Co-curated and organised by students, this event helped increase awareness on campus, provided students with a safe platform to join in the conversation on academic integrity issues and debate policies and procedures. The positive impact of such campaigns were recorded in publications such as Khan et al. (2020a), (2020b) and others.

### Tracking UOWD's response to COVID19 crisis—Teaching and Learning

Now that we have provided an overview of some of the longitudinal changes the University brought about to develop a campus of integrity, we will focus on the phase from 2020 when the COVID19 pandemic hit.

#### Distance learning sub committee

UOWD's transition to EDL due to the COVID19 pandemic was aided significantly due to prior planning for an institution-wide transition to blended learning that had commenced in January 2019. It is important to note here that the primary provider has an offshore partner campus in Hong Kong (UOW, n.d) where mass protests forced shutdowns and disruptions in delivering courses (Sun 2019; Lai 2019).

As a campus that plans ahead and considers all facets of governance and practice, the University recognised the need to address growing trends of students connecting with the digital space and role of technology enhancing teaching and learning (Garrison & Kanuka 2004; FitzGerald et al. 2013; Brown 2016; Lim & Wang 2016; Lalima & Dangwal 2017). The planning for blended learning that began in 2019 was invaluable as leadership and faculty had already started to implement the strategic vision, considering their learning design and assessment components. Therefore, when EDL was required in 2020, UOWD was already prepared to shift focus to the online modality of blended learning, making appropriate technological investment and offering professional development to faculty.

The already-formed Blended Learning Steering Committee adapted to form a dynamic task force for EDL. The Distance Learning subcommittee's agenda was to be agile (Yap et al. 2014; Betta & Owczarzak-Skomra 2019) in its decision-making to help empower faculty and guide the implementation of EDL across the institution, including the adaptation of assessment to suit the online modality across the institution, and to ensure a quality and consistent student experience in the online modality.

The Distance Learning subcommittee (DLS), chaired by the University President, consisted of members from senior leadership including:

- Deans and ADEs from each Faculty area,
- Directors of Academic Governance and IT from the University’s primary provider, and
- an education specialist in the field of blended and digital education

and maintained close collaboration with the Academic Registrar, faculty, and the quality assurance department (Gigliotti, 2019).

Studies have shown that building a robust digital capacity can help enhance students’ learning experience, engaging capacity with content and aid in developing a culture of integrity (Azevedo 2012; Fang 2012; Deranek & Panther 2015). The DLS was responsible for disseminating and implementing best practice in distance education, not just during EDL brought on by the COVID19 pandemic but prior to it, in addition to building digital capacity across the organisation through the minimum standard of LMS (learning management system) design, usage, content and student engagement, in alignment with the strategic vision of the University. The digital capacity building specified the effective use of the LMS, including outlining the mandated requirement that all faculty use the LMS and the expectations of how it was to be employed in each teaching session; and to provide (i) support and improve subject coordination, (ii) clear student orientation and scaffolding, and (iii) parity of experience across all subjects.

As sporadic distance education continues well into 2022 (the time of writing this paper), the committee’s agenda matures to further focus on quality assurance and enhancing the student experience by ensuring that UOWD provides a consistent, high-quality education experience.

In addition to online workshops for new students on Academic Integrity and Writing during orientation period, DLS also rolled out virtual training sessions for students that were run every semester to prepare them for remote exams and enhance their understanding of the values and importance of integrity.

#### Professional development

**Fig. 4:**
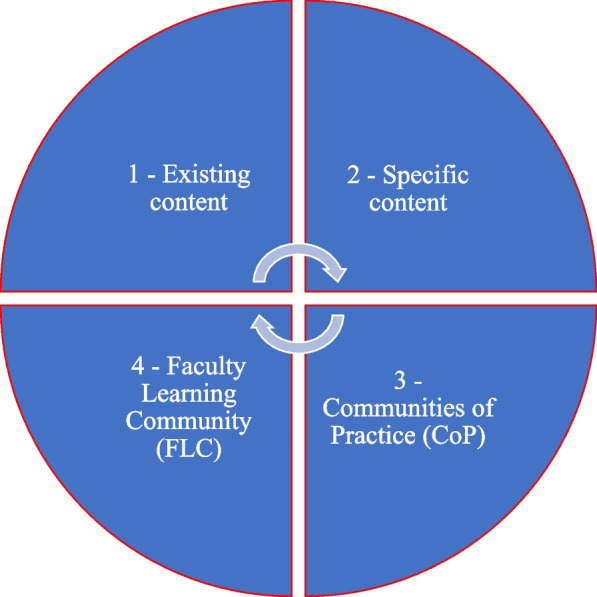
Matrix of offerings for professional development at UOWD

The long-term development of UOWD relates to the adoption of a heutagogical approach to professional development across the institution. The principles of heutagogy are providing ‘just-in-time’ support and ‘just in case’ training activities (Blaschke 2012). The concept is designed around the belief that recognises that people learn when they are ready and that this is most likely to occur quite randomly, chaotically and in the face of ambiguity and need (Blaschke 2012; Hase & Kenyon 2000). These principles were integrated into the design of the professional development during the pandemic as shown in Fig. 4, offerings of which took a number of forms: from leveraging existing resources, and new resources developed to enhancing online teaching practice as discussed below.

1. Existing content—such as LMS information packs and best practice examples that already existed were made available
2. Specific content – primary provider Learning & Teaching department offered self-paced (asynchronous) courses on Designing Teaching Online and Designing Online Assessment. Some of these were new, some were pre-existing
3. Communities of Practice (CoP) – informal communities of practice emerged across UOWD with learning champions from across the faculties supporting each other in technological and pedagogical approaches to remote learning (Wenger 2011). Faculty of Business and Faculty of Engineering & Information Sciences conducted their own sessions on academic misconducts through ad hoc academic blended learning teams who held practical sessions on how to set up exams virtually with training on online tools to use and how to use the online system for detection, using existing and specific content
4. Faculty Learning Community (FLC) – senior leadership worked to formalise and sustain the CoP by providing training to foster a more focused FLC across the institution. FLC’s foster connections between educators, to provide a collaborative community that supports each other on pedagogical issues (Cox 2004). Training was provided in the form of an intensive, interactive Bootcamp on Designing Distance education. The workshops focused on pedagogical and technical aspects and aimed to create collaboration across discipline areas during and beyond the delivery of the training, sometimes producing documentations that added to existing content databases.

#### Teaching & learning

As posited by Krishnamurthi & Rhode (2018), management needs to provide guidance and encouragement to faculty on matters such as course design and innovations to help prevent academic integrity issues. ADE in each faculty of the University was responsible for fostering an environment in which high-quality teaching and learning was valued, including through facilitating appropriate adoptions of new initiatives such as blended learning, work-integrated learning and use of technologies and contemporary pedagogical approaches in program delivery across the faculty.

Studies have shown teacher enthusiasm, attitude and proactive methods in teaching and learning can help reduce students’ likelihood to cheat (Khan 2014; Orosz et al. 2015). Similarly, du Rocher (2020) posited that active learning strategies can have significant positive influence on students’ attitude, deterring potential misconducts. Lecturers across the University got students to engage in different types of teaching and learning activities such as discussion forums, online quizzes, blogs, projects, breakout sessions, groups projects (outside of classroom hours), student presentations, problem solving online etc. These types of engagement led students to take more active roles in their own learning journey, and helping their fellow classmates, answering queries and engaging in discussions amongst themselves.

The learning analytics and student-learning interface was provided through the LMS at UOWD. To ensure the appropriateness and fitness for the purpose of LMS for this function, the University wrote a policy titled “Minimum Standards for the Use of LMS”. Use of learning analytics reports, and the LMS analytics report provided insight into the levels of student engagement in the programs. LMS analytics reports were provided to the faculty at the end of the third week of the semester to look at intervention strategies for engaging students (including contacting students). An end-of-semester report was also provided; this showed the level of student engagement which helped lecturers reach out and provide additional support to students (Amigud et al. 2019).

Laboratories were essential to engineering courses to meet learning outcomes. Studies have shown how STEM students were able to use file sharing websites to find solutions (Lancaster & Cotarlan 2021) and that those needed lab experiments were negatively impacted by EDL (Supernek, Ramirez & Supernek 2021; Hysaj & Suleymanova 2021). With Covid19, two strategies were employed by the University:

- The first was with regards to large physical equipment—this involved rethinking the delivery using either live recording or pre-recording of the laboratory but with instructors interacting online with the students, asking questions and explaining the experiment. Students were then required to write up the experiment based on the results collected by the instructors. These measures have been shown to have positive outcome in ensuring learning of concepts for students and deterring likelihood of misconducts (Grodotzki et al. 2021)
- Studies have also highlighted that being able to create an immersive environment to support student learning, stimulating higher order thinking beyond just theoretical concepts to actual applications, can reduce student motivation to cheat (Dhawan 2021; Dispeisse 2018). Therefore, the second approach by the University was to develop alternative software supported simulation or online simulators. For example, in motor drives, we were able to use Simulink, which the University provided to all students, to simulate more detailed aspects of the physical experiments as with Simulink it is possible to see non-measurable variables whereas in the physical one must derive them later by calculation. As of the time of writing this paper, the University is also in the process of developing a suite of “portable physical labs” (PP labs) that the University will post to students with instructors supervising them online. This is critical to help students develop practical design skills in engineering. For example, in embedded systems design, we sent each student a PP lab, and also provided them with the software development environment that they could download from a University server. This is a unique approach as the University is not aiming to be a distance learning University but one that adopts technology for a blended learning approach beyond the pandemic’s EDL.

Other efforts included:

1. All text-submissions were made through the text-matching software (embedded within LMS) to check for possible cases of plagiarism.
2. AIOs conducted workshops with faculty to provide information about possible situations of academic dishonesty, with case studies presented from global news media and research publications and how to report cases using an online system.
3. Alleged misconduct cases identified in assessments were reported to the Disciplinary Committee.

### Tracking UOWD's response to the pandemic—assessments

The ADEs reviewed all changes to assessment to ensure their suitability for distance learning during the pandemic. As part of the review, faculty members were provided with feedback on the design of their assessments to ensure suitability for online modes of delivery. Furthermore, as mentioned in the previous sections, the University already had a comprehensive list of policies and frameworks to support continuous improvement in teaching and learning at UOWD which further made it easy for the DLS committee to use as a base to work with, develop checklists, design informative training sessions – all of which helped agile decision making during crisis (Yap et al. 2014; Betta & Owczarzak-Skomra 2019) because they did not have to start from the beginning. As an example, the *Teaching and Assessment: Assessment and Feedback* policy document provides detailed guidelines on the design of assessments. The policy, for instance, specifies that the design of assessment tasks should support the development of the skills necessary to demonstrate academic integrity and minimise opportunities for academic misconduct. In alignment with this policy, the DLS approved the processes detailed below to ensure the rigour and integrity of assessments across all faculties.

#### Prior to assessments

1. Guidance documents (minimum requirements related to subject adjustments for remote delivery) were prepared by the external QA assurors (Ministry). The documents provided to QA assurors included changes to teaching and learning activities, assessments, minimum performance requirements (in alignment with Ministry directives) and an assessment of the impact on learning outcomes (if any). All these changes were approved at the various committees as well as discussed with and approved by the QA assurors at the primary provider campus. Students were also informed of the adjustment to the subjects.
2. Through a rigorous QA process internal to the faculties, lecturers ensured that security-checks were factored into the preparations of assignments for every subject module. These measures considered the augmented potential for students to source contract-cheating, plagiarise, utilise online search for answers or copy from each other’s work during Distance Learning (DL) Mode.
3. A set of rules (based on existing assessment policy) were developed to mitigate academic misconduct in online assessments. These included guidelines on how lecturers could create online assignments, the type of assessment measures such as incorporation of viva to validate students’ work (which existed for some, but not most subjects prior to EDL).

#### Assessment changes

1. Changes to assessments for all subjects underwent a rigorous QA process to ensure that learning outcomes are being assessed. For instance, most of the midterms were replaced by a few timed-online quizzes through online platforms. Although some of these were allowed to be in the form of multiple-choice questions, most were encouraged to be formative, rather than summative (Nguyen, Keuseman & Humston 2020; Noorbehbahani et al. 2022)
2. Changes to assessments included design of projects in stages, the use of mini case scenarios in MCQs (multiple choice questions) so that students could justify their chosen option, use of Online LMS Quiz format that allowed for randomisation of questions and further randomization alternatives during synchronous exams. LMS Quiz format allowed lecturers to set the time per each question to deter the students from checking exam answers through outside sources. Lecturers were also asked to refrain from using readily available questions from the publisher databases, test banks or online resources. Lecturers designed numerical questions that require explanation. For case-studies, for example, focusing on assigning themes specific to course content and time limited (announced within a tighter timeframe to mitigate chances of collusion or online search for answers).
3. Keeping in line with existing policies, guidelines were developed specifically to ensure consistency in the design of the various types of STEM assessments (synchronous and asynchronous) such as programming assignments, lab reports, individual and group reports, quizzes and exams which were all being conducted online.

#### Grading and monitoring changes

1. Staggered or cumulative grading was implemented, where a design/artefact/creative project was checked along several stages and the student was not able to submit the entire work at one go. As students would be doing online exams where they had access to resources, it was felt that exam questions needed to measure higher order thinking rather than definitional questions, to ensure students did not get additional advantage from open book exams. This was effective as students were required to demonstrate originality in their answers and thus reduced collaboration or direct reference to resources and in line with existing literature (Brookhart 2010; Khan et al. 2021; Wehlhurg 2021).
2. Depending on the nature of the subject, the following methods were used to ensure authenticity of student work: use of webcam (as per Ministry directive in the country), multiple versions of exams, practice sessions in using technology, use of drafts, rigorous cross check measures with previous work and random viva sessions with students (including suspected cases).
3. In the case of open book and asynchronous exams, faculty were directed to develop questions that measured higher order thinking, testing application, evaluation, and creation of knowledge. All exam papers went through a process of three tier QA based on checklists that were created by DLS based on existing policies and procedures. The first level was done by the subject QA assuror who primarily looked at ensuring the subject learning outcomes were met; second level by the Program Directors who primarily checked to see that the degree program’s outcomes were met, and final QA done by ADE who checked to ensure the DLS guidelines were followed. These additional levels of QA allowed evaluation of exam questions more closely to see they are targeted at higher levels of learning.
4. In 2020, the university explored proctoring services and concluded that it was not aligned to the University’s values i.e., respect for students and their privacy, understanding digital gap among students having access to fast Internet connection, students coming from various cultural and religious backgrounds, etc. Proctoring software use went beyond using webcams to lock and track browsers, keystrokes, use artificial intelligence to track students’ eye and lip movements, body movements, store such data and more making them more invasive in nature (Koops 2020).

### Measuring effectiveness of UOWD's response to pandemic

To gauge the effectiveness of UOWD's sudden shift to DL in helping to mitigate students’ likelihood to cheat, we were provided with a feedback report from the QA unit to identify the best practices and areas for improvement as well as areas of training and gaps in resources. The feedback report included summary results of subject evaluations, lecturer evaluations, EDL evaluations, student and faculty feedback collected after every semester and so on without any identifying details of subjects, students, units or faculty. The feedback report was developed by the QA unit in response to the authors’ queries on overall statistics and comments by stakeholders such as students and faculty in relation to such satisfaction and evaluation survey results, and any significant changes the QA unit noted from the DLS in recognition of such results. The QA unit then prepared a feedback report and sent it to us for use. This process received the ethical clearance and custodian permission.

#### Findings 1

a Upon reviewing the report, we found that, although for many it was a new experience, students were quickly adapting to the online mode.
  We attribute this level of ease and confidence in students to the University’s early efforts in moving to blended learning on campus as has been explained in previous semesters which would have introduced students to the digital platforms, process of attending online classes and taking online quizzes.
b Challenges faced by students included:
  a connectivity,
  b technical issues,
  c social connection,
  d practical tasks,
  e lab work,
  f class size and ability to participate,
  g use of LMS,
  h stress about submissions,
  i initial issues related to interactivity of classes, and
  j Initial issues related to participation

DLS recognised that some or most of these challenges were urgent to respond to as studies have found that such issues can act as catalysts for student misconducts (Valizadeh 2021; Noorbehbahani et al. 2022).

#### Findings 2

- The feedback report also showed the following were recognised as measures that helped alleviate some of the issues identified in Findings 1:
  - increased availability of lecturers online,
  - the increased use of discussion forums and
  - break-out rooms

DLS actions taken included:

- providing training and orientation sessions to students on the various technologies used,
- providing training and orientation sessions to students on change in learning and teaching design to make sessions more engaging and dynamic with provisions for instant feedback
- LMS sites were quality assured and faculty were provided with support to help organise their LMS sites to make them easier for students to navigate.
- an eLearning course was developed and delivered to students during orientation, outlining how to succeed in distance education from wellbeing and studying perspective.
- a comprehensive Digital Literacies course was designed to cover: Learning how to learn, Innovative approaches to learning, online learning strategies and digital wellbeing.

Existing literature has posited that such measures are key to ensuring likelihood of misconduct behaviours are deterred and that a holistic culture of integrity is set (Meccawy, Meccawy & Alsobhi, 2021).

#### Findings 3

The results of the semesters indicated that students were more comfortable with the mode of delivery and the satisfaction increased by 20% from semester to semester. Anonymous check-in surveys were also conducted at the beginning of the semester and actions were taken within week five of classes, the half point in the Subject delivery.

This finding is in alignment with Khalil et al. (2020) who posited that “online classes were well-accepted by the…students” (p. 1). Studies such as Jordan (2001), Nora and Zhang (2010) and Khan (2014) have suggested that intrinsic motivations and confidence can deter students from engaging in misconduct. So, this result is significant in pointing to the effectiveness of the measurements put in place to help students transition to EDL during pandemic, thus minimizing possibility of engaging in cheating behaviours.

#### Findings 4

- The results of the anonymised faculty survey indicated a change in the perception of the faculty in their confidence level prior to and after having gone through the experience of distant learning. For example, less than 60% of faculty were confident in moving towards distant mode. However, upon completion of the semester, the increase in confidence level was increased to above 90%.

b. Challenges faced by faculty included:
  - technical/connectivity issues, and
  - student participation/engagement.
  - the need to have more training in the areas of delivery tools, assessment design, online student engagement and curriculum design.

Özüdoğru (2021), Almahasees, Mohsen and Amin (2021) and Khan et al. (2022) have all indicated similar challenges faced b faculty during EDL.

#### Findings 5

- Higher engagement with the quieter students
- Improved attendance
- Collaborative environment between students
- Increased, efficient and flexible consultation hours
- Effective class sizes
- Effective assessment strategies
- Increased student accessibility to learning and teaching materials during class

All the changes and support systems placed by the University had a significant impact on managing misconducts and upholding integrity during remote teaching which is discussed further in the next section. It is important to note here that while proactive actions such as training, policies, and clear guidelines, etc. are important to develop a holistic culture of integrity, the other part of that equation is detection and penalty. Detection especially plays a significant role in helping to pave the way for learning opportunities that can also lead to rehabilitation and restoration of the alleged students (Perkins, Gezgin, and Roe, 2020; Cavalcanti et al 2012). Therefore, we focus on the detection data of the University across 2018—2020 to track the effectiveness of the COVID19 response by the University below as evidence.

### Measuring effectiveness: cases reported on the system

**Fig. 5:**
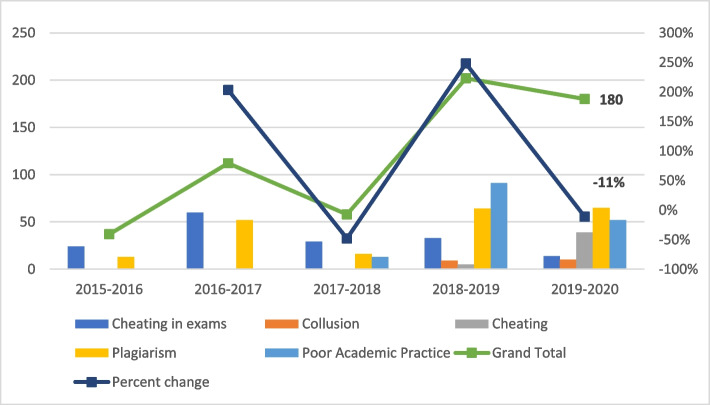
Historical data of cases reported—as summarised and provided by QA unit

Looking at a time series data from 2015 to 2020 (see Fig. 5), calculating the percentage changes, we discuss the following observations. The graph reflects the change in reported alleged cases on the system to track the trend over a period of years from 2015—2020. It is also showing the total for each time period and the percentage change in total cases between the time periods as shared in the feedback report by QA unit.

#### Observation 1

Historically, incidences directly related to exam and plagiarism were regularly reported, however without any significant pattern over the years. This seems to be in line with some literature that either focuses on, reports, or posits the likelihood of cheating in exams or plagiarism in assessments (McCabe, Butterfield &Trevino 2017; Middleearth 2014). In fact, early workshops, and training sessions with faculty at the University also revealed how they were able to detect plagiarism due to the use of the text-matching software used by the University, while the Registrars was in charge of training invigilators who in turn were diligent about reporting any disciplinary issues during exams or midterm exams.

#### Observation 2

It is very interesting to see that since the efforts of the University increased through awareness programs, workshops and other initiatives, the number of poor practice and collusion incidents were being reported (see Observation 5 and 6). Furthermore, faculty developed understanding of the different types of misconduct, nature and behaviour that defined those misconducts and how to detect them. This finding supports what has been posited in earlier studies such as Bjelobaba (2020) and Vuckovic et al. (2020).

#### Observation 3

While the rate of reporting of poor practice cases increased, historically, exam cheating incidents reduced; in fact, there was an 11% drop in cases reported in 2020. This is a rather interesting finding. While globally, institutions claimed greater cases of cheating in online exams, at this University, the total instances seemed to drop. Studies such as Dendir and Maxwell (2020), Janke et al. (2021) and others have postulated how students did in fact cheat more online. However, in this case study, there was a record drop of 11% in such cases. This anomaly may perhaps be explained by revisiting the continued efforts by the University in not only detecting cases, but also changing assessments, training faculty to re-design exam questions, formats, encouraging students with academic integrity pledges prior to taking online exams, and so on, all of which have been known to help develop a culture of integrity (Harrison 2020; Holden et al. 2021). Furthermore, it could also be that as the academic integrity policy matured, with more awareness and understanding of different forms of misconduct behaviours explained in the policy, not everything was reported as cheating (also see Observation 5, 6). It is crucial to note here the percentage changes recorded were per total number of students sitting for exams per year, negating the impact of reducing and removing exams in some instances during EDL on the actual percentage change.

#### Observation 4

Cheating as a broad category became popularly reported in 2019 and 2020. Based on informal discussions, it is posited this category largely included cheating in online quizzes, assessments, peers using group chats, websites for help, acquiring solutions and answers. This goes back to Observation 2 that posits the awareness among faculty to report more cases, and possibly to Observation 5 on collusion cases increasing around the same period (discussed further in sections below). We note here that a major role playing here as an external influence also could be the Australian government’s initiatives to introduce new laws to ban and punish essay mills which began making headlines in 2018–9, through to 2020 (Ross 2018; Hare 2019; McKie 2020). As a partner university for an Australian primary provider, these headlines coincided with the University’s efforts on the ground in organising the Week of Actions against Academic Misconduct, increased attendance and presenting of research findings, grants funding such projects and more (as has been described before in detail). It is not surprising then that overall awareness of faculty and push from management to ensure reporting of allegations would have an impact on the percentage of reports compared to previous years. Similar observations were made by Department of Education and Department of Employment and Workplace Relations under the Australian Government (DESE 2021; Awdry & Newton 2019).

#### Observation 5

Collusion is defined as any collaboration that is not allowed, as per the University’s policy. This was an interesting type of misconduct that showed up in the reporting from 2018–2019 which was not reported in earlier years, echoing Li et al. (2021) and Observation 2. Again, perhaps as the policy matured, the number of awareness programs and faculty training increased, AIOs were more involved, this would explain the inclusion collusion as a misconduct being selected by faculty when reporting.

#### Observation 6

Poor Academic Practice is defined as an action that is not necessarily deemed as misconduct, however at the same time reflects poor practice in academic writing. There was a significant increase in reported cases of poor practice in 2018–2019 from previous years. We may attribute this to the fact that the new policy, which was introduced in 2016, came into effect by 2017; therefore, increasing faculty awareness of this category. Furthermore, as the University moved away from punitive approach to educative approach, our informal discussions with faculty pointed to a possibility that faculty felt with low-level plagiarism cases with 1–2% text matching and attempts by students at citation with website links but quotations missing were now being reported by faculty as poor practice as it seemed less punitive for “mild” and unintentional cases. This is supported by findings from Pincus & Schmelkin (2003) that “faculty do not perceive academic dishonesty dichotomously as an all or nothing situation” (p. 206) and Keener et al. (2019) who posited that “faculty… assigned what they felt to be appropriate consequences directly based on their values and perceptions” (p. 4) which is related to their clarity of definitions (Pincus & Schmelkin, 2003).

However, what is more interesting is that there was a decrease in reporting poor practice in 2019–2020. This phenomenon could point to two possibilities:

- there were less alleged poor practice cases because students were now online and had access to more resources both allowed by the University in the form of open-book assessments, and others such as text-generators and other digital text modification. This is supported by existing literature as well (Gorenko, n.d.; Meuschke and Gipp 2013; Sakamoto & Tsuda 2019).
- there were less alleged poor practice cases because the actions were being identified as misconducts. However, given the evidence that faculty were demonstrating deeper understanding of types of misconduct, it is posited that the reason may very well be the first, that there were significantly more resources available which has also been detailed by UNESCO (2020).

#### Observation 7

Overall, the total number of cases reported increased prior to the pandemic but reduced during the pandemic. While at face value, this might seem strange and an anomaly, studies such as Harris et al. (2020) state that students are no more likely to cheat online when compared to face to face; or Harrison (2020) that suggests authentic assessments can indeed minimise students’ likelihood to cheat do make us ponder if all has been doom and gloom; or rather that such case studies as the University in this paper do shed light on the efforts made not just during the pandemic but over a decade to help develop a culture of integrity on campus that could very well explain this result.

It is important to note here that our findings remain inconclusive, thus pose certain limitations, on whether the actual cases and incidences of misconduct increased, decreased, or remained the same during the pandemic, echoing Holden et al. (2020) as we look at percentage of increase or decrease, rather than actual figures.

### Measuring effectiveness—faculty reporting

**Fig. 6:**
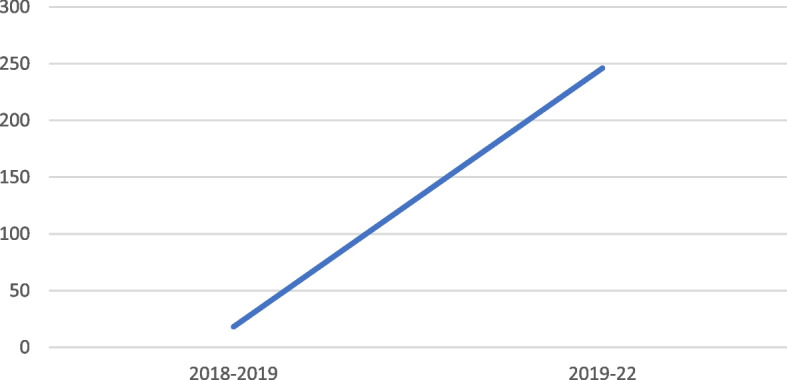
Tracking faculty reporting of alleged cases of misconduct

As mentioned previously, the reporting system was fully implemented by 2017. Through awareness workshops, flash cards and online instructional videos, the University was informing and training the faculty to use the system. As seen in Fig. 6, between 2018 to 2019, there was an 18.18% increase in the overall number of faculty members across all departments using the reporting system to report cases. However, between 2019 to 2020, this figure jumped to reflect a 246.15% increase!

It may be important to note here that as we were looking at percentage change, this figure demonstrates that although the total number of cases may have reduced in some places or for some categories, overall, there was more faculty reporting, rather than the handful of common, aware faculty who historically reported cases across all departments (as we discovered from the QA unit).

For instance, let’s assume x = number of faculty who reported cases prior to 2019.

If x = 5, given the percentage of increase (z) shown in Fig. 6, y = total number of faculty who reported cases in 2019 and 2020.

Using percentage increase formula: the $\mathrm{y}=\mathrm{x}+\left(\mathrm{z}*\mathrm{x}\right)$

The new value =

$$\begin{array}{l} 5+ \left(250{\% }\times 5\right)=\\ 5+250{\% }\times 5 =\\ \left(1+250{\%}\right) \times 5=\\ \left(100{\%}+250{\%}\right) \times 5=\\ 350{\% }\times 5=\\ 350\div100 \times 5=\\ 350\times 5\div100=\\ {1,750}\div100=\\ \boldsymbol{17.5} \end{array}$$

The above is an example of a possible calculation to demonstrate how the ‘percentage increase’ works. This can also help to explain how although in Fig. 5, percentage change in the number of reported cases dropped, however, the number of cases being reported included more faculty than previous years. Moreover, we understand this to reflect the increase in overall number of faculty using the system to log an allegation, which did not necessarily convert to an actual allegation and case, which might also justify the discrepancy between the two figures.

During workshops and informal faculty meetings, we observed faculty’s comfort in using the system and how they had become proactive in reporting any alleged cases they suspected during online exams or assessing.

Unlike studies like Merkel (2021) or Ray (2020) that posit there may be confusion surrounding academic integrity policy and procedures pertaining to misconducts committed online, our university’s years of efforts and continued work during the pandemic to address these have proven to support faculty members and the process of reporting, detecting, penalising and rehabilitating students, supporting findings from Coalter, Lim and Wanorie (2007).

Furthermore, during support sessions, it was observed that although faculty members voiced they thought it may be easier for students to cheat in online assessment, something that can also be seen in Lederman (2020); they also accepted that support provided by the faculties during workshops in understanding assessment design, restructuring and exam format were key to hindering misconduct behaviour by students, mirroring Harper et al. (2020) and Curtis et al. (2021).

### Key takeaways and overall guide

Now that we have highlighted the significant positive impact of changes made over a decade at the University which we believe helped the University uphold academic integrity, even during EDL, in this section we provide a guideline for any university as a reference and good practice guide for the future:

#### University governance members as stakeholders

- Identify and recognize academic integrity as a core value of the university
- Proactively engage in discussions on academic integrity, assessment practices, research scholarship and change management, not as biproduct of other discussions
- Identify all stakeholders across all levels and departments crucial to successfully developing a culture of integrity on campus as partners. These include but are not limited to—students, teachers/faculty (full time, part time, casual), administrative, professional staff (library, registrars, student services), greater community
- Regularly visit, review and revise policies and procedures
- Ensure to provide support for all stakeholders in understanding and implementing policies and procedures
- Address concerns and issues head on instead of ignoring or burying them
- Encourage and support research scholarship in academic integrity through events, grants, conferences, and publications
- Respond to external catalysts, even if they are across borders with a mind to focusing on upholding academic integrity
- Reflect annually on practices and outcomes by developing clear measures of success

#### University faculty as stakeholders

- Appreciate the academic integrity policy and procedures in place, ask for clarifications where something feels ambiguous, for instance a definition, a step to follow for reporting, or a process of applying penalty
- Attend awareness programs, trainings, and workshops to learn about the procedures, how they work
- Understand own role in subject delivery, assessment design and grading to uphold academic integrity
- Determine own role in detection and penalty as a necessary step to upholding integrity, ensuring transparency and fairness for all students
- Develop a scholarship of teaching and learning practice with view to situate academic integrity in this discourse
- Follow national and international news pertaining to academic integrity issues
- Be a role model in research and teaching and learning with integrity
- Be proactive
- Ask for support

#### University students as stakeholders

- Attend awareness programs, trainings, and workshops to develop understanding of university’s positionality on academic integrity
- Develop clear understanding on the values of academic integrity
- Develop clear understanding of the policies and procedures in place on coursework, academic integrity, and misconducts
- Understand the various behaviours that may constitute a misconduct
- Practice academic integrity values in academic life
- Learn about legitimate sources and resources available from university
- Become a champion for the academic integrity values and spread the message to other students
- Ask for clarifications and support when in doubt

## Conclusion

The pandemic has made it difficult for learning to continue for students who were previously attending face to face models of teaching and learning. While higher education institutions have battled to keep their virtual doors open, the question of integrity has become an ever-insistent elephant in the room. Studies such as Lancaster and Cotarlan (2021) have posited how there has been a sharp increase in the number of students accessing and approaching essay mills during online exams, although there is still little or no research to show that cheating has increased during the pandemic.

This paper has attempted to research and understand one university’s response to the pandemic and how it has worked to uphold integrity during emergency distance learning.

A western University campus in the Middle East, UOWD has been championing academic integrity research, funding projects, conducting training and outreach efforts since 2006. The systematic efforts by the University long before the pandemic, that forced it to adopt emergency distance learning, sowed the seed for building a culture of integrity among its students and faculty much before the pandemic hit.

This paper traces the journey of the University to recognise the challenges that its students and faculty faced, and how the University brought about reforms, policies and procedures that were more proactive than reactive in helping the University make agile decisions during crisis and transition to EDL and still maintain integrity.

The case study shows a decade-long effort that the University made, sometimes triggered by external events such as the Australia’s MyMaster scandal, with collaboration from various departments and stakeholders in bringing about changes to subject restructuring, assessment design and formats for exams. The University regularly supported and highlighted research, funded projects, published news and hosted conferences on academic integrity, not only involving its own students and faculty, but also working to create communities of practice locally and regionally, successfully showcasing implementation of the 4 M (mega-macro-meso-micro) framework as described by Eaton (2021).

From setting up special task groups, to empowering faculty to conduct formal and informal training, to holding town hall meetings with students, the University adopted a holistic approach to managing EDL, ensuring both students and faculty were supported in a manner that would help to encourage and uphold integrity. While the University does not claim that their efforts have ensured they had no misconducts, authors posit that indeed the efforts by the University supported the faculty and they felt safe and encouraged to become vigilant and use the academic integrity online system to both detect and report allegations. The percentage changes in the number of cases of allegations reported also point to the fact that the training, workshops, information, videos, and the general culture of the University that was already pre-existent, helped detect and report such cases, thus perhaps acting as further deterrent to the actual misconduct behaviours (Cavalcanti et al 2012).

Using a reflective case methodology, this paper aimed only to highlight the University response as a possible good practice guide that demonstrates the importance of having a culture of integrity on campus which can help to weather crises such as EDL during the COVID19 pandemic.

***The authors do not claim generalisation of any form or kind and only report the University's efforts and responses with due permission from custodians of the data presented in the study***.

## Data Availability

All data is available upon request.

## Appendix

Table 1.

**Table 1:** List of some publications related to academic integrity by faculty and staff at University of Wollongong in Dubai

No	Authors	Title	Publication	Year
1	Khan, Zeenath;	E-Learning And E-Cheating: Drawing A Balance	Internal White Paper	2007
2	Khan, Zeenath Reza; Samuel, Stephen D;	E-Cheating, Online Sources And Technologies: A Critical Review Of Existing Literature	Conference Paper	2009
3	Khan, Zeenath;	E-Cheating And Calculator Technology: A Preliminary Study Into Casual Implications Of Calculator-Technology Usage On Students' Attitude Toward E-Cheating	Conference Paper	2009
4	Khan, Zeenath Reza;	E-Cheating In The UAE: A Critical Review Of Existing Literature	Conference Paper	2010
5	Khan, Zeenath Reza;	E-Cheating And Calculator-Technology	Technological Developments In Networking, Education And Automation	2010
6	Khan, Zeenath Reza;	E-Cheating And Societal Factors-A Critical Review Of Existing Literature	Conference Paper	2010
7	Khan, Zeenath Reza;	Technology Use Takes A Boost Among University Students	Journal Article	2012
8	Khan, Zeenath Reza; Balasubramanian, Sreejith;	Students Go Click, Flick And Cheat… E-Cheating, Technologies And More	Journal Of Academic And Business Ethics	2012
9	Khan, Zeenath Reza; Balasubramanian, Sreejith;	Libraries Opt For More Online Sources	Innovations And Advances In Computer, Information, Systems Sciences, And Engineering	2013
10	Khan, Zeenath Reza;	Plagiarism-Tracking Software: Instructors' Perception Of Software Use Vs. Actual Purpose Of Software	Conference Paper	2013
11	Khan, Zeenath Reza;	Software Use Vs. Actual Purpose Of Software	Tarc International Conference On Learning And Teaching	2013
12	Reza Khan, Zeenath;	Developing A Factor-Model To Understand The Impact Of Factors On Higher Education Students’ Likelihood To E-Cheat	Doctoral Thesis	2014
13	Khan, ZR;	"Exploring Emerging Academic Integrity Issues In Education Sectors"—Proceedings International Conference On Academic Integrity Middle East Chapter (Ed)	International Conference On Academic Integrity—Middle East Chapter 2016, Dubai, UAE, University Of Wollongong In Dubai	2016
14	Khan, Zeenath Reza; Mumtaz, Sabiha; Harish, Priyanka; Raheja, Sanjana;	Pilot Study To Pave Way For Exploring Contract Cheating Among Higher Education Students In UAE	4th International Conference Plagiarism Across Europe And Beyond 2018, ENAI, Turkey, Pp. 76–76	2018
15	Khan, Zeenath Reza; Khelalfa, Halim; Sarabdeen, Jawahitha; Harish, Priyanka; Raheja, Sanjana;	Paving The Way For An Higher Ed Academic Integrity Policy Review In The UAE	4th International Conference Plagiarism Across Europe And Beyond 2018, ENAI, Turkey	2018
16	Sabiha Mumtaz, Wardah QURESHI, Eman ABU EL RUB	Explaining Differential Cheating Behavior Of Business Vs. Medical Students	4th International Conference Plagiarism Across Europe And Beyond 2018, ENAI, Turkey	2018
17	Khan, Zeenath Reza;	What Category Are They Anyway?: Proposing A New Taxonomy For Factors That May Influence Students' Likelihood To E-Cheat	Scholarly Ethics And Publishing: Breakthroughs In Research And Practice	2019
18	Khan, Zeenath Reza; Mumtaz, Sabiha; Hemnani, Priyanka; Raheja, Sanjana;	Whose Work Is It Anyway? Exploring The Existence Of Contract Cheating In The UAE Context	Towards Consistency And Transparency In Academic Integrity	2019
19	Zeenath Reza, KHAN; HEMNANI, Priyanka; RAHEJA, Sanjana; JOSHY, Jefin;	Awareness Programs Against Contract Cheating At A Middle Eastern University–Pathway To Building Campus-Wide Culture Of Integrity	5th International Conference Plagairism Across Europe And Beyond	2019
20	Zeenath Reza, KHAN; MUMTAZ, Sabiha; RAKHMAN, Salma Sadia;	Tracing The Journey Of Two Students’ Trajectory To Becoming Advocates Of Integrity–A Case Study	5th International Conference Plagairism Across Europe And Beyond	2019
21	Khan, Zeenath Reza; Khelalfa, Halim; Sarabdeen, Jawahitha; Harish, Priyanka; Raheja, Sanjana;	Preliminary Review-Universities’ Open Source Academic Integrity Policies In The UAE	Proceedings Of The 2019 International Conference On Frontiers In Education: Computer Science & Computer Engineering, CSREA Press, United States	2019
22	Gaizauskaite, Inga; Sivasubramaniam, Shivadas; Glendinning, Irene; Razi, Salim; Bjelobaba, Sonja; Kralikova, Veronika; Khan, Zeenath Reza Khanzeenath Reza;	Surveying Academic Integrity: Methodological Issues And Lessons Learned	5th International Conference Plagiarism Across Europe And Beyond & 3rd International Conference Shaping Ethics In Academia And Society At: Vilnius, Lithuania	2019
23	Khan, Zeenath Reza; Priyanka; Raheja, Sanjana; Joshy, Jefin;	Raising Awareness On Contract Cheating –Lessons Learned From Running Campus-Wide Campaigns	Journal Of Academic Ethics	2020
24	Khan, Zeenath Reza; Mumtaz, Sabiha; Rakhman, Salma Sadia;	Mentoring To Affect Student Perceptions Of Academic Integrity	Academic Language And Learning Support Services In Higher Education	2020
25	Rakhman, Salma Sadia; Khan, Zeenath Reza;	Equity In Admission: Comparative Study Of Secondary Data In High School Curriculum Valuation	6th International Conference Plagiarism Across Europe And Beyond 2020	2020
26	Khan, Zeenath Reza; Dyer, UAE Jarret; Bjelobaba, Sonja; Gomes, Sandra F; Dlabolova, Dita; Sivasubramaniam, Shivadas;	Gamifying Academic Integrity–The First Steps	6th International Conference Plagiarism Across Europe And Beyond 2020	2020
27	Venugopal, Swathi; Khan, Zeenath Reza;	Journey From Classroom To Workplace–One Student’s Story	Integrity In Education For Future Happiness	2020
28	(Ed) Khan, Zeenath Reza; Hill, Christopher; Foltynek, Tomas;	Integrity In Education For Future Happiness	Edited Book	2020
29	Khan, Z. R; Mulani, V.;	Contract Cheating Values In School Assessments – What Values Are We Really Teaching Our Young Students?	6th International Conference Plagiarism Across Europe And Beyond 2020	2020
30	Khan, Zeenath Reza; Sharma, Vidhi; Thomas, Tina;	Ai Triage – Helping Faltering Students Rehabilitate From Academic Misconducts	Integrity In Education For Future Happiness	2020
31	Rakhman, Salma Sadia; Khan, Zeenath Reza;	Equity In Admission—A Comparative Study Of Secondary Data In High School Curriculum Valuation	Integrity In Education For Future Happiness	2020
32	Heather Lea Harvey, Sanjai K Parahoo, Sabiha Mumtaz, Darwish Badran, Kamal Banihani	Investigating Individual And Situational Factors Influencing Academic Integrity: An Empirical Study Among Medical Students	Educational Alternatives	2020
33	Hysaj, A. And Elkhouly, A., 2020	Why Do Students Plagiarize? The Case Of Multicultural Students In The United Arab Emirates	In ENAI Conference	2020
34	Hysaj, Ajrina, And Doaa Hamam	Academic Writing Skills In The Online Platform-A Success, A Failure Or Something In Between?	*In IEEE International Conference On Teaching, Assessment, And Learning For Engineering (TALE), Pp. 668–673*	2020
35	Hysaj, Ajrina, And Sara Suleymanova	The Analysis Of Developing The Application Of Critical Thinking In Oral And Written Discussions	*In IEEE International Conference On Teaching, Assessment, And Learning For Engineering (TALE), Pp. 819–824*	2020
36	Khan, Zeenath Reza; Dyer, Jarret; Bjelobaba, Sonja; Gomes, Sandra F; Dlabolová, Dita Henek; Sivasubramaniam, Shivadas; Biju, Soly Mathew; Hysaj, Ajrina; Harish, Priyanka;	Initiating Count Down-Gamification Of Academic Integrity	International Journal For Educational Integrity	2021
37	Khan, Zeenath Reza; Mulani, Veena;	Managing Academic Integrity In Primary School Assessments By Managing Parental Involvement	European Conference On Academic Integrity And Plagiarism 2021	2021
38	Khan, Zeenath Reza; Hysaj, Ajrina; John, Serene Regi; Khan, Sara;	Gateway To Preparing K-12 Students For Higher Education–Reflections On Organizing An Academic Integrity Camp	European Conference On Academic Integrity And Plagiarism 2021	2021
39	Khan, Zeenath Reza; Sivasubramaniam, Shiva D; Anand, Pranit; Hysaj, Ajrina;	The Role E-Tools Play In Supporting Teaching And Assessments With Integrity During The Covid-19 Pandemic	European Conference On Academic Integrity And Plagiarism 2021	2021
40	Vel, Prakash; Khan, Zeenath Reza;	Contract Cheating Incidents In Schools And Tertiary Learning Institutions In The Uae From A Social Lens	European Conference On Academic Integrity And Plagiarism 2021	2021
41	Hill, Christopher; Khan, Zeenath Reza;	Calling Out The Elephant In The Room: Integrity And Ethical Practices In Times Of Crises–Experience From The Middle-East	European Conference On Academic Integrity And Plagiarism 2021	2021
42	Khan, Zeenath; Arvikar, Atharv; Venugopal, Swathi; Hemnani, Priyanka;	Corporate Plagiarism During Remote Work–A Concern?	Canadian Symposium On Academic Integrity, June 23—24 2021, Thompson Rivers University	2021
43	Sivasubramaniam, Shivadas; Dlabolová, Dita Henek; Kralikova, Veronika; Khan, Zeenath Reza;	Assisting You To Advance With Ethics In Research: An Introduction To Ethical Governance And Application Procedures	International Journal For Educational Integrity	2021
44	Khan, Zeenath Reza; Sivasubramaniam, Shivadas; Anand, Pranit; Hysaj, Ajrina;	‘E’-Thinking Teaching And Assessment To Uphold Academic Integrity: Lessons Learned From Emergency Distance Learning	International Journal For Educational Integrity	2021
45	Razi, Salim; Sivasubramaniam, Shiva; Eaton, Sarah Elaine; Bryukhovetska, Olha; Glendinning, Irene; Khan, Zeenath Reza; Bjelobaba, Sonja; Çelik, Özgür; Topkaya, Ece Zehir;	Systematic Collaboration To Promote Academic Integrity During Emergency Crisis	Canadian Perspectives On Academic Integrity	2021
46	Khan, Zeenath Reza; Sivasubramaniam, Shivadas; Gomes, Sandra F; Bjelobaba, Sonja; Razi, Salim; Waddington, Lorna; Ribeiro, Laura;	Gamification Of Academic Integrity: Reviewing An Evaluation Tool	Canadian Perspectives On Academic Integrity	2021
47	Khan, Zeenath Reza; Draper, Michael; Bjelobaba, Sonja; Razı, Salim; Sivasubramaniam, Shiva, D.;	Making Academic Integrity Accessible The Outreach Way	European Conference On Academic Integrity And Plagiarism 2021	2021
48	Sabiha Mumtaz, Sabreen Wahbeh, Eman Abuelrub	Academic Integrity In Online Exams: An Exploratory Study Of Student Perceptions In The Uae	European Conference On Academic Integrity And Plagiarism 2021	2021
49	Hysaj, A And Suleymanova	Safeguarding Academic Integrity In Crisis Induced Environment: A Case Study Of Emirati Engineering And Computer Science Students	In International Conference On Human–Computer Interaction (Pp. 236–245). Springer, Cham	2021
50	Hysaj, A	COVID-19 Pandemic And Online Teaching From The Lenses Of K-12 STEM Teachers In Albania	*In 2021 IEEE International Conference On Engineering, Technology & Education (TALE) (Pp. 01–07)*	2021
51	Khan, Zeenath Reza; Balasubramanian, Sreejith;	An ISM Approach To Modeling: Antecedents Of E-Cheating In Higher Education	Research Anthology On Interventions In Student Behavior And Misconduct	2022
52	Razı, Salim; Glendinning, Irene; Eaton, Sarah Elaine; Çelik, Özgür; Khan, Zeenath Reza; Bjelobaba, Sonja; Fishman, Teddi; Waddington, Lorna;	Changing Trends In Academic Integrity Policy Development: Implications For The Post-COVID Era	Conference Paper	2022
53	Gomathi Kadayam Guruswami, Sabiha Mumtaz, Aji Gopakumar, Engila Khan, Fatima Abdullah, Sanjai K Parahoo	Academic Integrity Perceptions Among Health-Professions’ Students: A Cross-Sectional Study In The Middle East	Journal Of Academic Ethics	2022
54	Hysaj, A., Freeman, M. And Khan, Z.R.,	Teaching Academic Writing Skills: A Narrative Literature Review Of Unifying Academic Values	In ENAI Conference	2022

## Abbreviations

EDL: Emergency distance learning
US: United States of America
UAE: United Arab Emirates
MoE: UAE Ministry of Education
UOWD: University of Wollongong in Dubai
QA: Quality assurance
USAID: United States Agency for International Development
UOW: University of Wollongong Australia
CAA: Commission for Academic Accreditation
TEQSA: Tertiary Education Quality and Standards Agency Australia
AIO: Academic Integrity Office
WAAAM: Week of Actions Against Academic Misconduct
DLS: Distance learning sub-committee
ADE: Associate Dean for Education
UOWGE: University of Wollongong Global Enterprise
LMS: Learning management system
CoP: Communities of Practice
FLC: Faculty learning community
DL: Distance learning
MCQs: Multiple choice questions
4 M: Mega-macro-meso-micro
STEM: Science, technology, engineering, mathematics
PP: Portable physical
